# Association of Blood Cell Parameters of Peripheral Inflammation With Brain Imaging Measures

**DOI:** 10.1093/geroni/igab046.2433

**Published:** 2021-12-17

**Authors:** Yuan Fang, Kathryn Lunetta, Jesse Mez, Michael Alosco, Claudia Satizabal, Wendy Qiu, Margaret Doyle, Joanne Murabito

**Affiliations:** 1 Boston University School of Public Health, Boston, Massachusetts, United States; 2 Boston University, Boston, Massachusetts, United States; 3 Glenn Biggs Institute for Alzheimer’s & Neurodegenerative Diseases, University of Texas Health Science Center, San Antonio, Texas, United States; 4 University of Vermont, Colchester, Vermont, United States; 5 Boston University, Framingham, Massachusetts, United States

## Abstract

Neutrophil to lymphocyte ratio (NLR), red cell distribution width (RDW), and mean platelet volume (MPV), are easily measured circulating blood cell parameters that reflect chronic peripheral inflammation which increases risk for dementia and Alzheimer’s disease (AD). We investigated the cross-sectional association between these blood cell parameters and brain MRI measures, including total cerebral brain volume (TCBV) as percentage of total intracranial volume (TCV) to correct for differences in head size, hippocampal volume (HPV) and log transformed white matter hyperintensity (WMH) volume, in the Framingham Heart Study (FHS) cohorts. We identified 2882 FHS participants 25 to 92 years of age (mean 59 years), 53% women, who attended an exam that included a complete blood cell count sample and received a brain MRI within five years of blood draw. We used linear mixed effect models to examine associations, adjusting for age, age^2, sex, education, cohort, time between blood draw and MRI, prevalent cardiovascular disease, C-reactive protein, APOE-ϵ4 genotype and TCV for HPV and WMH, and accounting for familial correlation using a random effect. We observed significant (p≤0.01) associations between higher RDW and smaller TCBV, and between elevated NLR and larger WMH volume. Analysis on an older subgroup (age ≥60 years, mean 71 years, n=1357) demonstrated larger effect sizes and additional significance between increased RDW with smaller HPV. We conclude that chronic peripheral inflammation as measured by NLR and RDW associates with MRI measures of brain aging (TCBV, HPV) and vascular brain injury (WMH) in FHS, with stronger impact in participants ≥60 years.

